# Obesity and cancer—extracellular matrix, angiogenesis, and adrenergic signaling as unusual suspects linking the two diseases

**DOI:** 10.1007/s10555-022-10058-y

**Published:** 2022-09-08

**Authors:** Natalia S. Pellegata, Mauricio Berriel Diaz, Maria Rohm, Stephan Herzig

**Affiliations:** 1 Institute for Diabetes and Cancer, Helmholtz Center Munich, 85764 Neuherberg, Germany; 2 Joint Heidelberg-IDC Translational Diabetes Program, Inner Medicine 1, Heidelberg University Hospital, Heidelberg, Germany; 3 German Center for Diabetes Research (DZD), 85764 Neuherberg, Germany; 4 Chair Molecular Metabolic Control, Technical University Munich, Munich, Germany

**Keywords:** Cancer, Obesity, Angiogenesis, Fibrosis, Adrenergic signaling

## Abstract

Obesity is an established risk factor for several human cancers. Given the association between excess body weight and cancer, the increasing rates of obesity worldwide are worrisome. A variety of obesity-related factors has been implicated in cancer initiation, progression, and response to therapy. These factors include circulating nutritional factors, hormones, and cytokines, causing hyperinsulinemia, inflammation, and adipose tissue dysfunction. The impact of these conditions on cancer development and progression has been the focus of extensive literature. In this review, we concentrate on processes that can link obesity and cancer, and which provide a novel perspective: extracellular matrix remodeling, angiogenesis, and adrenergic signaling. We describe molecular mechanisms involved in these processes, which represent putative targets for intervention. Liver, pancreas, and breast cancers were chosen as exemplary disease models. In view of the expanding epidemic of obesity, a better understanding of the tumorigenic process in obese individuals might lead to more effective treatments and preventive measures.

## Introduction

Obesity is a well-established risk factor for several human malignancies including hepatocellular carcinoma (HCC), pancreatic adenocarcinoma (PDAC), and breast cancer, among others [9]. We selected these three cancers as primary disease models for our review. Hepatocellular carcinoma (HCC) is the 6th most commonly diagnosed cancer and the second most common cause of cancer-related deaths worldwide [27], with a 5-year survival rate of 18% in the USA (source: ACS 2022). Typically, liver cancer results from a series of pathological changes, which progress into fibrosis and subsequent cirrhosis, and ultimately to HCC. While traditionally linked to hepatitis B/C infection, HCC has been increasingly associated with metabolic diseases such as the metabolic syndrome or type 2 diabetes mellitus (T2DM), which often co-occur with non-alcoholic fatty liver disease (NAFLD) or non-alcoholic steatohepatitis (NASH) [5]. This link is predicted to cause an increasing cancer occurrence in the population in the near future [64]. Pancreatic ductal adenocarcinoma (PDAC) is the 12^th^ most common cancer worldwide but is the 4^th^ most frequent cause of cancer-related deaths. PDAC has the highest mortality rate of all major cancers, with a 5-year relative survival rate of only 11% (source: SEER database 2022). Efforts have been made to elucidate the early events promoting the neoplastic transformation of pancreatic cells. Acinar-to-ductal reprogramming is a key event in cancer initiation, and acinar-to-ductal metaplasia (ADM) lesions represent the earliest pre-neoplastic lesions predisposing to PDAC [115]. Pancreatic intraepithelial neoplasia (PanIN), the most common precursor lesion for PDAC, can arise from ADM. Large epidemiological studies have demonstrated that metabolic disorders such as obesity, insulin resistance, and T2DM increase the risk of developing PDAC [249].

Breast cancer is the most common invasive malignancy in women worldwide, and the second leading cause of cancer-related deaths in women (source: ACS 2022). The overall 5-year survival rate for breast cancer is 90%, with differences however depending on whether the disease is localized (up to almost 99%) or associated with metastatic spread (< 30%). Postmenopausal breast cancer is 1.2- to 1.4-fold more likely in patients affected by overweight or obesity [169].

In addition to the cancer types discussed herein, many other cancers are also affected by body weight. Indeed, a large population-based cohort study demonstrated that 17 out of 22 cancer types were associated with body mass index (BMI), albeit not to the same extent [23].

There is compelling evidence that circulating nutritional factors (lipids, glucose), hormones (insulin, insulin-like growth factor, leptin), and cytokines (interleukins, tumor necrosis factor), among others, connect obesity and cancer. These have been discussed and reviewed in greater detail elsewhere and will not be the topic of this review. Instead, we here aim to describe novel potential links between elevated body weight and cancer through exploring common molecular pathways between the two diseases, considering common treatment approaches, and outlining possible connections. We highlight three potential links—extracellular matrix remodeling, angiogenesis, and adrenergic signaling—and discuss their roles in cancer, focusing on HCC, PDAC, and breast cancer.

## Extracellular matrix remodeling and fibrosis linking obesity and cancer

### Pathological fibrosis is a consequence of excessive ECM deposition

Pathological fibrosis (or fibrotic scarring) results from increased deposition of extracellular matrix (ECM) components such as collagens and fibronectin, leading to excessive formation of connective tissue replacing parenchymal tissue and affecting tissue architecture and normal organ function [89]. Fibrosis can develop in numerous organs as a consequence of uncontrolled regenerative processes in response to many types of repetitive injuries and chronic inflammation, which can progress to irreversible tissue scaring, tissue dysfunction, and eventually organ failure [172].

In different organs exhibiting pathological fibrosis, myofibroblasts are the main source of excessive ECM components [154]. Interestingly, different progenitor cells can acquire a myofibroblastic phenotype in response to pro-fibrotic signals such as transforming growth factor (TGF)β1, the latter representing the prototypical inducer of a SMAD2/3 transcription factors-mediated gene program driving myofibroblast differentiation [206]. Lineage tracing studies provided strong evidence indicating that myofibroblasts mainly originate from local mesenchymal cells [57, 130]. However, in renal, hepatic and pulmonary fibrosis, it has been suggested that myofibroblasts can arise from local epithelial (progenitor) cells undergoing epithelial-to-mesenchymal transition (EMT) [248]. Generally, myofibroblasts are characterized by mesenchymal properties such as proliferation and mobility, as well as the production and secretion of ECM components [127, 248]. De novo expression of fibroblastic α-smooth muscle actin (αSMA) in stress fibers is a hallmark of mature myofibroblasts, enabling their high contractile activity. Myofibroblasts arising from different tissue-specific sets of progenitors and distinct molecular mechanisms might dictate fibrosis-related processes contributing to both, metabolic disease progression and tumorigenesis, as well as respective etiological interconnections.

### ECM remodeling and fibrosis in cancer

More than 30 years ago, Harold Dvorak presented his view on tumors as “wounds that do not heal,” a concept emphasizing the analogy between tumor stroma generation and wound healing, which he still advocates after decades of research [59]. Originally, this concept was based on the identification of tumor-derived vascular endothelial growth factor (VEGF) and its role in initiating the formation of a vascularized connective tissue that solid tumors need to survive, grow and metastasize, which resembles that found in healing wounds [60].

In both wound healing and tumor development, fibroblast recruitment and activation are essential for ECM remodeling supporting tissue repair or tumorigenesis, respectively [189]. Similar to myofibroblasts, cancer-associated fibroblasts (CAFs) were originally identified as αSMA-positive cells characterized by a contractile phenotype and synthesis and secretion of ECM components. Notably, recent studies indicated the co-existence of distinct subsets of CAFs within the tumor microenvironment and an association of CAF heterogeneity with tumor (sub-) types as well as their pleiotropic tumorigenic functions [47, 114].

**Fig. 1:**
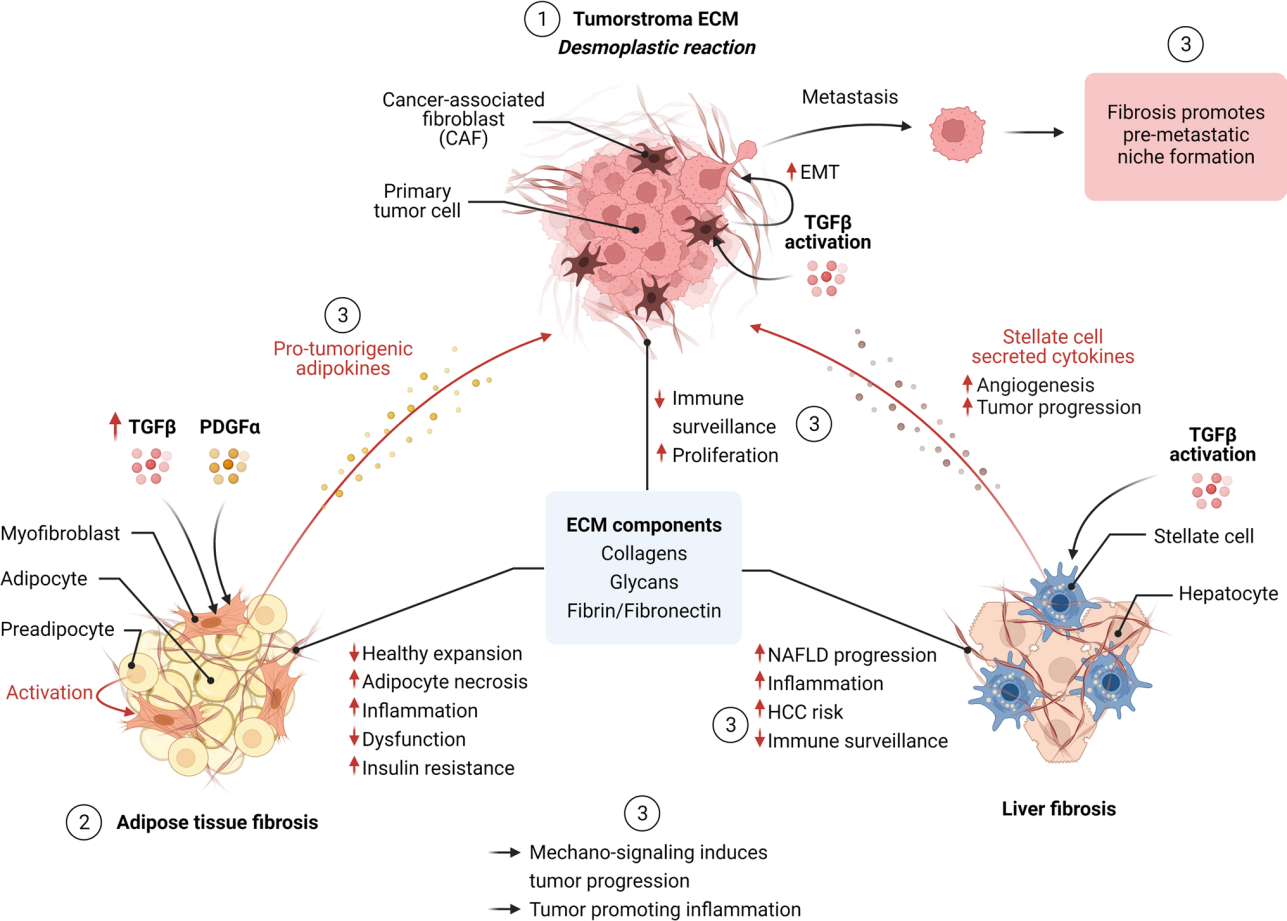
Overview of fibrosis in cancer and obesity. (1) ECM remodeling and fibrosis in cancer: The development of a fibrotic tumor stroma (desmoplastic reaction) is a multistep process in part driven by activation of cancer-associated fibroblasts (CAFs) and TGFbeta signaling. CAFs and ECM components exert different pro-tumorigenic processes including epithelial-mesenchymal transition (EMT) in tumor cells, thereby inducing tumor progression (metastasis formation). A fibrotic tumor stroma prevents immune cell infiltration, thereby reducing immune surveillance and responsiveness to cancer immunotherapy. (2) Adipose tissue fibrosis in obesity: Obesity-driven pathologic expansion of adipose tissue is characterized by adipocyte hypertrophy and cell death, chronic inflammation (including macrophage infiltration), and enhanced fibrosis. Fibrosis limits the expansion of adipose and contributes to metabolic dysfunction, including systemic inflammation and insulin resistance. Elevated TGFβ levels under obesity conditions contribute to adipose tissue fibrosis and inflammation. PDGF signaling induces a myofibroblast-like phenotype in a subset of adipocyte progenitors contributing to ECM deposition. (3) ECM remodeling and fibrosis linking metabolism and cancer: Mammary adipose tissue fibrosis contributes to malignant transformation and breast cancer progression through various mechanisms including collagen-derived endotrophin, increased tissue stiffness activating mechano-sensitive pathways, and induced inflammation (e.g., inducing a tumor-promoting macrophage phenotype). The degree of liver fibrosis is a strong predictor of NAFLD progression towards HCC. Activation of stellate cells (myofibroblast phenotype) by TGFβ and other cytokines mainly contributes to hepatic fibrosis. Stellate cell-secreted cytokines (including TGFbeta, PDGF, and VEGF) induce angiogenesis, reduced immune surveillance, and HCC progression. Obesity-induced pancreatic inflammation and desmoplasis contribute to PDAC progression and chemotherapy resistance

The development of a fibrotic tumor stroma is known as a desmoplastic reaction to a neoplasm, representing a dynamic and multistep process, the characteristics of which vary between different tumors [251]. The early phase of provisional matrix generation, composed primarily of fibrin and (to a lesser extent) fibronectin, plays a critical role in angiogenesis (in part induced by fibrin degradation products). It is associated with pro-tumorigenic inflammation and initial activation of resident and invading immune and other stromal cell populations such as perivascular cells, resident stem/progenitor cells, and quiescent fibroblasts. Subsequently, the increase in fibronectin deposition defines the late provisional ECM, which serves as a scaffold for growth factors and enables mechanical signaling, e.g., by representing a depot for TGFβ latent complex and contributing to additional differentiation of normal fibroblast to generate CAFs [93, 156]. The increasingly stiff fibronectin matrix promotes the deposition of collagen fibers produced by cancer-associated fibroblasts and the transition to a more mature state of the fibrotic tumor stroma [224, 251]. Pro-fibrotic cytokines, most importantly TGFβ, and reduced collagen degradation drive this process, resulting in fibrillary collagen accumulation and further stiffening of the tumor stroma (Fig. 1). Of note, the degree of ECM density and rigidity of tumor tissue, in addition to playing a role in cancer diagnosis, is an important factor of cancer progression and response to therapy due to changes in the biophysical properties and mechano-signaling [61, 210].

The central role of the tumor microenvironment (TME; or tumor stroma) in regulating various aspects of cancer biology is undisputed, although the complex and context-dependent interplay between different cell types and their activation status, ECM components, and corresponding upstream regulators is far from being fully understood. Cancer-associated fibroblasts (CAFs), which are present in high numbers in the TME, were shown to elicit various pro-tumorigenic functions. For example, both pre-malignant and malignant mammary epithelial cells adopted a mesenchymal phenotype and an induced metastatic potential when co-cultured with CAFs, whereas exposure to normal fibroblasts promoted an epithelial phenotype and suppressed metastasis formation [58]. Interestingly, this study showed that the capacity of CAFs to induce a mesenchymal-like phenotype (including mesenchymal morphology and induced marker expression) was at least in part mediated by distinct deposition of ECM components, the effect of which could be blocked in the presence of collagenase [58]. In another study on breast cancer, tumors were classified based on ECM composition. Strikingly, the resulting ECM subclasses were predictive of patient outcome, emphasizing the impact of the ECM components on tumor progression [18]. In preclinical models of lung cancer, induced expression of lysyl hydroxylases 2 (LH2) in CAFs induced a collagen cross-link switch in the tumor stroma, which in turn promoted the migratory and invasive properties of lung adenocarcinoma [181].

It has been proposed that the dense tumor stroma induced by the CAFs can represent a barrier preventing efficient immune cell infiltration, thereby contributing to impaired immune surveillance and supporting tumor growth [205]. In agreement, a large pan-cancer analysis defined a distinct set of ECM upregulated genes associated with worse prognosis that was correlated with activated TGFβ signaling in CAFs and immunosuppression [36]. Indeed, another study in patients with metastatic urothelial cancer treated with anti-PD-L1 agent atezolizumab demonstrated that lack of therapy response was associated with a signature of TGFβ signaling in fibroblasts [155]. Treatment unresponsive tumors were characterized by exclusion of CD8( +) T cells from the tumor parenchyma that were located in the fibroblast- and collagen-rich peritumoral stroma. Interestingly, in a mouse model recapitulating the immune-excluding CD8( +) T phenotype, T-cell penetration, and tumor regression was induced by co-administration of TGFβ-blocking and anti-PD-L1 antibodies, indicating that TGFβ shapes the tumor microenvironment to restrain anti-tumor immunity [155]. Conversely, it remains unclear to which extent (or in which specific context) treatments resulting in ECM remodeling could induce the release of growth factors and cytokines that in turn could promote tumor growth and unrestrained dissemination of tumor cells. However, re-educating the TME to exert anti-tumorigenic functions represents a promising approach as an anti-cancer therapy, particularly in combination with immune checkpoint inhibition [193]. However, despite the promising effects of pharmacological targeting of TGFβ signaling for anti-cancer therapy in cell culture and animal models, anti-TGFβ therapies resulted in poor or inconsistent outcomes in cancer clinical trials [234]. There might be various reasons underlying the discrepancies between preclinical and clinical treatment outcomes, in particular, preclinical models only insufficiently reflect the cancers in patients (e.g., with respect to treatment timing and cancer stage). In addition, inappropriate patient selection in clinical trials, as well as the complexity and pleiotropic nature of TGFβ functions might contribute to the poor clinical translation. Moreover, targeting of TGFβ with various approaches mainly aimed at inhibiting cancer cell invasion and metastasis is associated with specific challenges concerning clinical study design and treatment endpoints in regard to demonstrating survival benefits [4]. Therefore, blocking the immunosuppressive function of TGFβ in combination with established immune checkpoint inhibitors, as exemplified above [155], might improve the outcome of clinical studies.

### Adipose tissue fibrosis in obesity

Adipose tissue is classified as either white, brown, or beige/brite based on whether it functions as an energy store or thermogenic organ. The white adipose tissue stores energy in the form of triglycerides in lipid droplets, and it is composed of adipocytes (40–50% of cells) but also of connective tissue matrix, neural cells, and non-adipocyte cells (e.g. preadipocytes, immune cells, endothelial cells) that constitute the stromal vascular fraction (SVF) [204]. Obesity-driven pathological expansion of adipose tissue is characterized by adipocyte hypertrophy (increased cell size) accompanied by hypoxia (due to insufficient angiogenesis to support tissue growth), a state of chronic inflammation, and enhanced fibrosis [132] (Fig. 1). In contrast, a high capacity for adipocyte hyperplasia (increased cell number upon pre-adipocyte recruitment) is metabolically more favorable, enabling “healthy” expansion of adipose tissue [218]. Enlarged adipocytes exhibit different necrosis-like abnormalities contributing to inflammation by promoting elevated infiltrating of pro-inflammatory M1-polarized adipose tissue macrophages (ATMs) and other immune cells, as well as reducing the levels of M2-polarized anti-inflammatory ATMs and regulatory T cells (Tregs) [132, 154]. However, there are contradicting findings concerning the functional phenotype of adipose tissue macrophages. Studies using human adipose tissue samples suggested that obesity is associated with enriched M2-polarized macrophages [83, 258]. Moreover, there might be spatiotemporal differences in the origin and distribution of adipose tissue macrophages under obesity conditions [150]. Still, a state of unresolved chronic inflammation and formation of crown-like structures is considered a central driver of pathological tissue remodeling including the development of adipose tissue fibrosis [154].

Pioneering studies in mice demonstrated that the expression of different collagens in adipose tissue, the latter representing major ECM components, is induced under metabolically challenging conditions such as obesity and diabetes [120]. Particularly the enhanced deposition of type VI collagen represents a hallmark of adipose tissue fibrosis, limiting the expandability of adipose tissue and impairing the capacity to store the surplus of lipids appropriately as obesity progresses. Dietary and genetic mouse models of obesity lacking type VI collagen (Col6) displayed increased adipose tissue storing capacity, which was associated with reduced ectopic lipid accumulation in non-adipose tissues such as the liver, and overall improved systemic glucose and lipid homeostasis [120]. The concept that ECM accumulation represents a physical constrain to healthy adipose tissue expansion and plasticity, and thereby aggravates metabolic dysfunction, was corroborated by further studies. Induced expression of Col6 was confirmed in adipose tissue of obese humans, resulting in dysfunctional ECM that was associated with increased adiposity and chronic inflammation [186]. Conversely, inhibition of lysyl oxidase (LOX), an enzyme cross-linking collagen types I and III to form the fibrillary collagen fibers, ameliorated metabolic dysfunction and adipose tissue inflammation in obese mice [85]. In the same study, the authors proposed a model in which adipose tissue hypoxia serves as an early upstream initiator for adipose tissue dysfunction by inducing a local state of fibrosis. They found increased hypoxia and induced nuclear protein levels of the transcription factor hypoxia-inducible factor (HIF-) 1α in adipose tissue from obese (ob/ob) mice in comparison with lean wild-type mice. Notably, adipose tissue-specific overexpression of a constitutively active form of the HIF-1α in mice failed to induce an angiogenic response but resulted in increased adipose tissue fibrosis as well as local inflammation [85].

Besides the contribution of collagens to the structural remodeling of adipose tissue under metabolic conditions, ECM components play a role in disease-associated signaling. For instance, the adipokine endotrophin represents a carboxy-terminal cleavage product of collagen type VIα3 chain (Col6α3) and contributes to adipose tissue fibrosis and metabolic dysfunction [231]. Using mice with adipose tissue-specific overexpression of endotrophin, the study by Sun and colleagues demonstrated that endotrophin exerts local effects on the adipose tissue microenvironment, aggravating fibrosis and contributing to systemic inflammation and insulin resistance [231], the latter suggesting a potential role in the risk connection between metabolic diseases and cancer (see below).

As stated above, TGFβ represents an important pro-fibrotic signal, which, among its pleiotropic functions, induces myofibroblast differentiation from diverse precursor cell lines [206]. Notably, different studies demonstrated that both, circulating levels of TGFβ as well as TGFβ expression in adipose tissue are elevated under obesity conditions [242, 250]. Interestingly, the study by Vila et al. in obese mice suggested a direct link between metabolic endotoxemia (elevated bacterial lipopolysaccharide (LPS) levels), Toll-like receptor (TLR) 4-dependent macrophage activation, and induced adipose tissue fibrosis, which was widely prevented by antibody-mediated neutralization of TGFβ signaling [242]. Different studies showed that platelet-derived growth factor (PDGF) and signaling through PDGF receptor α (PDGFRα) importantly contributed to adipose tissue fibrosis [106, 153, 154]. In fibrotic adipose tissue, PDGFRα expressing (PDGFRα +) adipocyte progenitor cells adopted a myofibroblast-like phenotype expressing high levels of fibrosis markers such as collagens rather than differentiating to adipocytes [153]. Specifically, a subset of adipose tissue PDGFRα + progenitors with high levels of the surface marker CD9 (CH9hi) accumulated under pro-fibrotic conditions, and the concomitant phenotype switch towards myofibroblast differentiation was associated with increased ECM deposition and metabolic alterations including the development of insulin resistance [153]. These findings indicated that the composition of adipose tissue progenitor cell populations might affect the equilibrium between the adipogenic and the myofibroblast fate in response to pro-fibrotic signals [106, 153] (Fig. 1). The central role of liver fibrosis in NAFLD progression contributing to both, liver cirrhosis and HCC development is discussed in the next chapter.

### ECM remodeling and fibrosis linking metabolism and cancer

Various studies have provided evidence for the contribution of ECM remodeling in obese tissues to tumorigenesis, including breast, liver, and pancreatic cancer. As stated above, collagen type VI (Col6) strongly contributes to adipose tissue fibrosis under obesity conditions [120] and the Col6α3-derived carboxy-terminal cleavage product endotrophin further promotes adipose tissue fibrosis and metabolic dysfunction [231]. Interestingly, another study by the Scherer lab demonstrated that adipocyte-derived endotrophin augmented tumor growth and metastasis formation in the PyMT mouse model of breast cancer [183]. These findings added a fibrosis-related factor to the list of adipokines contributing to tumorigenesis in the context of obesity, such as leptin and others [22, 200]. Interestingly, endotrophin synergized with the canonical TGFβ pathway to promote EMT and lung metastasis [182]. A later study confirmed that endotrophin also contributes to breast cancer progression in humans, linking obesity to breast cancer aggressiveness and representing an interesting target for breast cancer therapy as well as anti-fibrotic treatments [28].

As exemplified by endotrophin, ECM remodeling–dependent alterations of adipose tissue endocrine functions and respective changes in adipokine and cytokine levels might exert pro-tumorigenic functions under obesity conditions. In addition, recent studies have pointed out that obesity-related changes in the structural properties of adipose tissue due to fibrosis could also contribute to the risk connection between obesity and cancer [56]. Indeed, diet and genetically induced obese mice displayed enhanced interstitial fibrosis and myofibroblast enrichment in mammary adipose tissue, suggesting a link between these established risk factors in breast cancer [213]. Interestingly, the seeding of breast cancer cells on decellularized matrices from obese adipose stromal cells stimulated their mechanosensitive growth and induced the malignant potential of pre-malignant human breast epithelial cells [213]. Moreover, the interaction of breast cancer cells with stiffness-promoting ECM components in mammary fat promoted tumor progression by inducing nuclear translocation of the transcription factor TWIST1, which in turn promoted EMT and metastasis formation [245]. The study deciphered a mechano-transduction pathway through which biomechanical properties of the TME alter tumor cell aggressiveness and possibly disease outcome.

The ECM remodeling in obesity could also have an impact on breast cancer through its effects on non-tumor cells of the TME. For example, a study using human breast cancer samples suggested that obesity increased the abundance of M2-polarized macrophages in breast adipose tissue, the effect of which was correlated with the degree of interstitial fibrosis [227]. The authors emphasized the notion that the phenotype of macrophages accumulating under obesity and fibrotic conditions were more similar to tumor-associated macrophages, and might therefore support tumor growth. A study by Hermano et al. proposed a different link between the tumor-promoting effects of the impact of obesity on ECM composition in estrogen receptor (ER)-positive breast cancer [90]. Interestingly, in mice deficient for heparanase, an endoglucuronidase that cleaves heparan sulfate in ECM, the acceleration of tumor progression in diet-induced obesity was abolished. The authors proposed a complex mechanism in which heparanase, which is preferentially expressed in obesity-associated breast tumors, promoted the secretion of inflammatory mediators by adipose tissue macrophages (TAMs), which induced local induction of aromatase, the latter representing a rate-limiting enzyme in estrogen biosynthesis. In turn, elevated estrogen levels further promoted heparanase production by ER-positive tumor cells, which contributed to the acquisition of a tumor-promoting phenotype of tumor-associated macrophages [90]. This study represents a good example of the complex mutual interplay of different cellular compartments contributing to the development of a specific cancer subtype. Besides its effects on the local environment of the primary tumor, obesity might render distant sites more susceptible to metastasis formation, the effect of which might also imply fibrotic processes. For example, obesity promoted the recruitment of myeloid lineage cells and induced the deposition of collagen fibers in the lung, thereby creating a microenvironment similar to described tumor-induced pre-metastatic niche and promoting metastasis formation [92].

Non-alcoholic fatty liver disease (NAFLD) represents the hepatic manifestation of the metabolic syndrome and encompasses different components of progressive liver disease ranging from hepatic steatosis (or non-alcoholic fatty liver (NAFL); without substantial hepatocellular injury) and non-alcoholic steatohepatitis (NASH; steatosis with inflammation and hepatocyte ballooning degeneration) to fibrosis, liver cirrhosis and hepatocellular carcinoma (HCC) [5] (Fig. 1). Only subsets of patients will progress along the sequence of worsened pathophysiological states from NAFL to NASH and further to cirrhosis, thereby being exposed to an increasing risk for HCC development [246]. The pathophysiological mechanisms underlying the risk connection between NAFLD and HCC include various components such as immune and inflammatory responses, DNA damage, oxidative stress, changes in the microbiome, and others that go beyond the scope of this review and have been comprehensively summarized elsewhere [5]. However, the degree of liver fibrosis has been shown to be the strongest predictor of overall and liver disease-specific mortality [62, 84], suggesting that this histological feature, at least in more advanced stages, might define irreversible states of progressive liver disease. Though, against the previous dogma that fibrosis is an irreversible process, other studies demonstrated that fibrosis can regress upon removal of the responsible driver, including weight reduction upon gastric band placing [54]. Besides the treatment of the underlying metabolic syndrome, therapies that would prevent or regress liver fibrosis could markedly reduce HCC and overall mortality risk in patients with progressive NAFLD. This notion is supported by a retrospective Korean study, in which the associations between features of NAFLD and HCC as well as other neoplastic diseases have been analyzed in more than 25.000 subjects over an average of 7.5 years [123, 124]. Strikingly, a high fibrosis score, rather than the degree of steatosis, was most strongly associated with the development of HCC as well as extrahepatic cancer. However, there is currently no approved antifibrotic pharmacotherapy for the treatment of NASH. Further understanding of the molecular mechanisms underlying NASH development might provide novel therapeutic targets in the future. For example, a recent study identified transcription factor networks directing intra-hepatic crosstalk between hepatocytes and stellate cells necessary for NASH and fibrosis progression [147]. Therefore, “regulatory hub-centered” targeting might provide novel strategies for the treatment of NASH.

Although different cells have been proposed to give rise to hepatic myofibroblasts through epithelial–mesenchymal transition (EMT), most studies suggest that hepatic stellate cells (HSC) are the main contributor to liver fibrosis in vivo [160, 168]. Upon progression of NAFLD to NASH, death of hepatocytes and cholangiocytes due to chronic liver injury causes activation of HSCs directly and through different inflammatory cytokines, chemokines, and growth factors released by resident Kupffer cells, infiltrating monocytes and other cell types [53]. Among other pro-fibrogenic factors, TGFβ represents a central player in the development of liver fibrosis by promoting the activation of HSCs into myofibroblasts and inducing the synthesis of ECM components such as type I and type II collagens [53, 55]. Additionally, activated HSCs can contribute to tumor development by secretion of cytokines and growth factors (including TGFβ, PDGF, and VEGF) inducing angiogenesis, reduced immune surveillance, and tumor progression [2, 53] (Fig. 1). Furthermore, liver fibrosis is a central feature of a pre-malignant environment favoring the development of liver cancer by various mechanisms [2]. Qualitative and quantitative changes in the ECM, besides affecting the structural properties of the tissue, have profound effects on cell signaling through direct interaction as well as the regulation of soluble growth factor activity [99]. Surrounding cells sense changes in the ECM by discoidin domain receptors (DDRs) and integrins that regulate intracellular signaling pathways and responsiveness to additional extracellular stimuli. For example, integrin activation can induce phosphoinositide 3 kinase (PI3Kinase) and mitogen-activated protein kinase (MAPK) signaling, both pathways with central roles in tumor development [260].

Obesity also significantly increases pancreatic cancer risk [74]. Similar to breast and liver cancer, fibrosis might contribute to pancreatic ductal adenocarcinoma (PDAC) development [102]. The study by Incio et al. demonstrated that obesity-induced pancreatic inflammation and desmoplasia contributed to PDAC progression and chemotherapy resistance [102]. The authors proposed a model in which adipocyte-derived IL-1β resulted in tumor-associated neutrophil recruitment which in turn activated pancreatic stellate cells. Besides several differences in the underlying molecular mechanisms, fibrotic and ECM remodeling processes might importantly contribute to obesity-driven cancer development in different tissues.

## Angiogenesis and angiogenesis-related proteins linking obesity and cancer

### Angiogenesis is tightly controlled through angiogenic factors

**Fig. 2:**
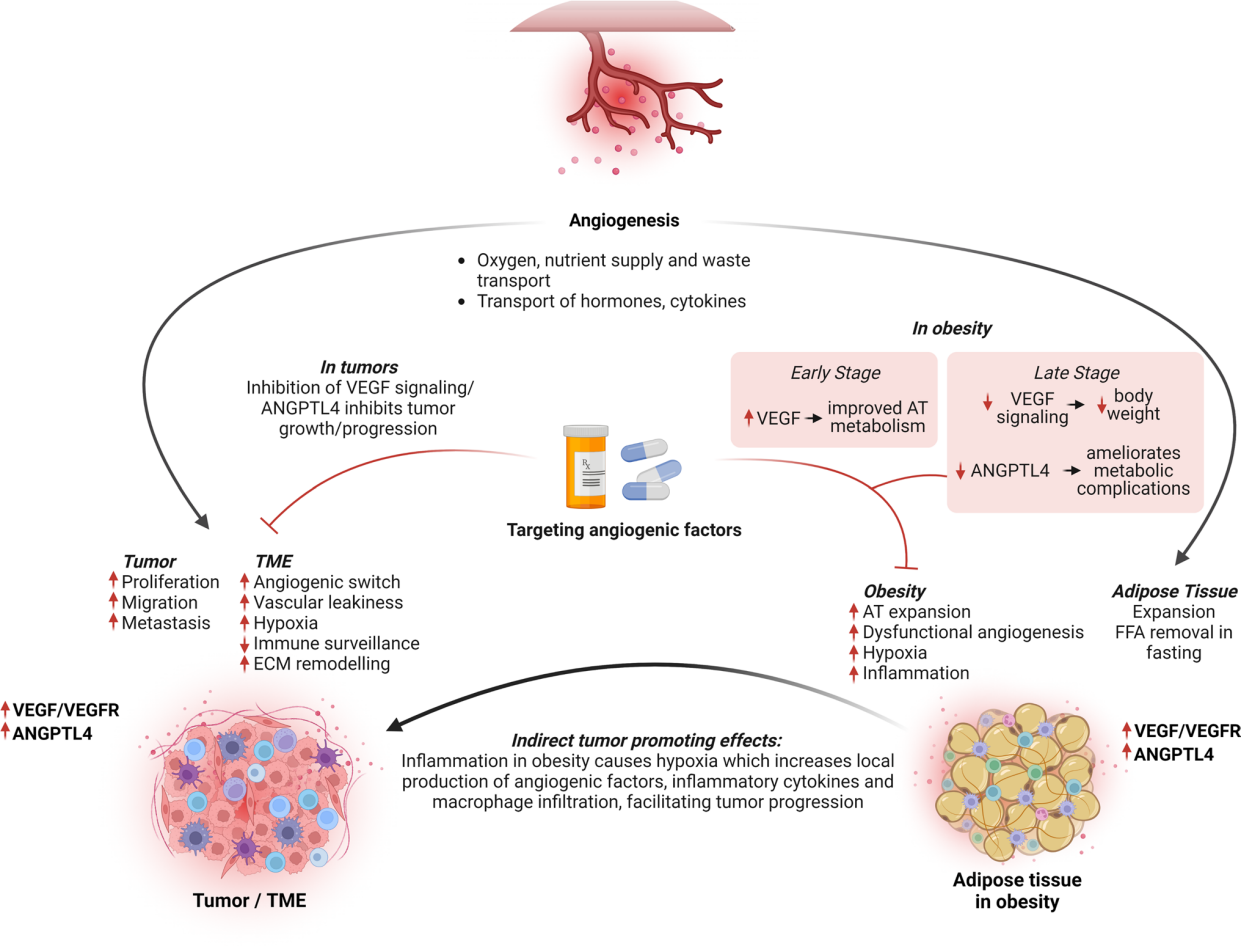
Angiogenesis and angiogenic signaling linking cancer and obesity. In the tumor microenvironment (TME), hypoxia induces pro-angiogenic signals, and the “angiogenic switch”, which in turn supports tumor growth, vascular leakiness, and extracellular matrix (ECM) remodeling. VEGF signaling and ANGPTL4 are important players in these processes and are associated with more aggressive tumor behavior. In obesity, the expansion of the adipose tissue (AT) leads to hypoxia, dysfunctional angiogenesis, inflammation, and elevated expression of VEGF and ANGPTL4. The obese AT secretes angiogenic factors and cytokines that promote tumor progression, including VEGF and ANGPTL4, which also act on the AT itself. Therefore, these two factors are an example of molecules at the crossroad between cancer and obesity, and represent targets for therapeutic intervention in both pathological conditions: inhibition of VEGF signaling and ANGPTL4 can inhibit tumor progression, and improve metabolic conditions in obesity, albeit in the latter the different role of VEGF in early and late disease stages needs to be taken into account

The term *angiogenesis* describes the sprouting of new capillaries from a pre-existing vasculature (Fig. 2). In adult tissues, vascular homeostasis is strictly controlled in both time and space by a variety of pro- and anti-angiogenic factors. When these factors are in balance, the vasculature is quiescent and endothelial cells do not proliferate. When pro-angiogenic signals prevail over anti-angiogenic signals, new blood vessel formation takes place. Physiological angiogenesis is stimulated by various conditions, including tissue ischemia, hypoxia, and inflammation. In hypoxia, there is a stabilization of the hypoxia-inducible factor (HIF)-1α, an oxygen-sensitive transcription factor, which in turn promotes the expression of numerous pro-angiogenic genes, such as *VEGFA*, encoding the vascular endothelial growth factor (VEGF), genes coding for VEGF receptors, and for angiopoietin-like 4 (ANGPTL4), among others [95].

#### Angiogenesis is a critical component of cancer initiation and progression

In tumorigenesis, early activation of angiogenic processes is mandatory to sustain the higher energetic demands associated with the enhanced proliferation of the tumor cells and the associated tumor tissue growth. As stated above, new blood vessel formation occurs when pro-angiogenic signals surpass anti-angiogenic signals, and in tumors, this process is termed the “angiogenic switch” [13]. The pioneering work of Douglas Hanahan and Judah Folkman using the *Rip*-*Tag* transgenic mice overexpressing the SV40 large T antigen in pancreatic β-cells established the role of the angiogenic switch as a rate-limiting factor sustaining tumor growth and progression [1, 68, 87]. The angiogenic switch promotes the growth of the malignant cells and allows the escape of tumors from dormancy. Thanks to Judah Folkman’s revolutionary hypothesis that blocking angiogenesis might stop tumor growth, two decades ago, anti-angiogenic therapy became a new modality in cancer treatment in addition to surgery, radio- and chemo-therapy.

Tumor angiogenesis is typically initiated from the capillaries and includes distinct cellular processes, including sprouting angiogenesis, intussusceptive angiogenesis, vasculogenesis, and transdifferentiation of cancer stem cells (reviewed in [149]). Mechanistically, activation of angiogenic processes involves the degradation of the vascular ECM, followed by endothelial cell proliferation and migration [263]. ECM remodeling is essential for the migration of endothelial cells and the formation of capillary sprouts, a process that requires the activation of matrix metalloproteases (MMPs), which degrade the basal membrane and the ECM. The *Rip*-*Tag* mouse model was instrumental in establishing a role for VEGF in the activation of ECM-degrading enzymes, thereby supporting the angiogenic switch [19, 112, 176] (Fig. 2).

Tumor angiogenesis results in the formation of poorly organized and malformed vascular networks, characterized by a loss of endothelial cell junctions. This increases both vessel permeabilization and interstitial pressure, which reduce tumor tissue perfusion. Hypoxia, the condition of low oxygen in tissues, is typically associated with the progression of solid tumors since with the expansion of the tumor mass, the distance between tumor cells and vessels increases, leading to local regions of poor oxygenation. Hypoxia is also a plausible cause of obesity-associated tissue inflammation (Fig. 2). Hypoxia is also a consequence of the malformed and dysfunctional vasculature and the low perfusion of the tumors. To overcome the limited oxygen and nutrient supply, hypoxic tumor cells upregulate the hypoxia-inducible factors, which then initiate pro-angiogenic programs by upregulating the transcription of downstream targets involved in angiogenesis, cell survival, and proliferation [197].

Noteworthy, in addition to serving as nutrient, oxygen, and waste transport providers, vessels also facilitate the dissemination of tumor cells to distant sites, thereby promoting metastasis. The development of metastases is the major cause of cancer morbidity and mortality and accounts for the vast majority (up to 90%) of cancer deaths [135].

#### Angiogenesis mediators and anti-angiogenic therapies in cancer

Hypoxia-mediated HIF activation increases the expression of *VEGFA* and *ANGPTL4*, among others. VEGFA is a member of a family of growth factors comprising also VEGFB, VEGFC, VEGFD, and PGF (placental growth factor), and functions as the main endothelial cell survival factor. It binds to two tyrosine kinase receptors, i.e., VEGF receptor 1 (VEGFR1) and receptor 2 (VEGFR2) and prompts their homo- and heterodimerization, transphosphorylation, and the activation of downstream signaling. VEGFA is primarily secreted by tumor and stromal cells in the TME and acts chiefly on endothelial cells by interacting with VEGFR2, the principal mediator of the cellular responses to the growth factor. VEGFR2 activation promotes endothelial cell proliferation, survival, and migration via the stimulation of PLCγ-PKC, MAPK-ERK1/2, and PI3K-AKT pathways [122, 232, 233, 244]. The function of VEGFR1 is less defined: it has been shown that a soluble version of VEGFR1 (sVEGFR1) is secreted by endothelial cells and can act as an endogenous decoy for VEGFA by sequestering it and blocking its access to VEGF receptors [105]. VEGFA also induces vascular permeability by activating mechanisms such as the phosphorylation of vascular-endothelial (VE)-cadherin and β-catenin, which cause the destabilization of endothelial cell–cell contacts and the opening of endothelial cell junctions [10]. Interestingly, adipocytes also produce VEGF and other pro-angiogenic factors (Fig. 2).

Given its central role in angiogenesis, VEGF signaling is upregulated in a variety of human cancers. The expression of VEGFR2 is elevated in HCC patients [8], as well as the levels of circulating VEGFA, which correlate with tumor angiogenesis, rapid disease progression, and decreased survival [209]. Based on these observations, therapies targeting the VEGFA pathway have been first evaluated in preclinical models of HCC, and then implemented in clinics with promising results [66, 143]. Sorafenib, a multikinase inhibitor targeting VEGFR, PDGFR, c-Kit, and RAF, has been the standard of care for patients with advanced, unresectable HCC for over a decade given its ability to increase patients’ survival [144]. Lenvatinib, another anti-angiogenic drug inhibiting VEGFR, fibroblast growth factor (FGF), and PDGFR with higher potency than sorafenib, is also used for unresectable HCC and showed an improvement in overall survival of the patients which was comparable to sorafenib [131]. Additionally, bevacizumab (a humanized monoclonal antibody that sequesters VEGFA) in combination with atezolizumab (immune checkpoint inhibitor) was approved by the FDA in 2020 for the treatment of patients with unresectable or metastatic HCC. Currently, this combination therapy is the first-line systemic therapy for such patients [67].

While HCC is a highly vascular tumor, with angiogenesis playing an important role in its growth and dissemination, PDAC exhibits poor vasculature, low blood flow, and reduced perfusion compared with normal pancreas. The resulting hypoxic environment further exacerbates pathological changes, such as the development of stromal fibrosis, a hallmark of PDAC. Tissue hypoxia promotes the release of various pro-angiogenic factors including VEGFA [261]. Accordingly, it was initially reported that PDAC tissues show an increase in *VEGFA* gene expression when compared to the normal human pancreas and that 60–65% of human PDAC samples show detectable VEGFA immunoreactivity[100, 104, 214]. However, recent RNA-Seq data (The Cancer Genome Atlas-TCGA dataset) challenged these findings given that only 8 out of 178 (4%) human PDAC samples were found to overexpress *VEGFA*, thereby suggesting that it may not be as relevant a player in PDAC tumorigenesis as firstly assumed [243]. Several genetically engineered mouse models of PDAC that successfully recapitulated the histological and molecular evolution of the tumors have been generated and proven to be instrumental in assessing the efficacy of drugs targeting the TME and angiogenesis. Among the tested drugs is sunitinib, a multi-kinase inhibitor targeting VEGFR and PDGFR signaling [178], which could not reduce tumor burden in PDAC mouse models. Clinical trials mirrored these results by showing a lack of efficacy of sunitinib when combined with gemcitabine for treating patients with advanced/metastatic PDAC[20], or when used as second-line therapy after failure to respond to gemcitabine [177]. Two additional anti-angiogenic drugs, bevacizumab and axitinib, also failed to improve the survival of PDAC patients [137]. Altogether, targeting VEGF signaling does not seem to represent a valuable strategy for the treatment of PDAC.

The interaction of VEGFA and VEGF receptors (VEGFR) and the resulting angiogenesis have been heavily implicated in breast cancer development, progression, and metastasis. High VEGFA and VEGFR expression in breast cancer patients correlates with worse outcomes and resistance to systemic therapy. Drugs with anti-angiogenic effects have shown beneficial effects in breast cancer patients, starting from tamoxifen, the mainstay adjuvant therapy for hormone-positive breast cancer. Originally believed to be a mere competitor of estradiol, tamoxifen was later found to inhibit VEGFA and angiogenesis [24, 72]. VEGFA is upregulated in breast cancers overexpressing the receptor tyrosine kinase human epidermal growth factor 2 (HER2). For these patients, the standard-of-care therapy is the anti-HER2 antibody trastuzumab alone or in combination with chemotherapy. Trastuzumab was shown to inhibit angiogenesis and normalize the tumor vasculature [107].

Another important mediator of angiogenesis is ANGPTL4, a secreted factor belonging to a superfamily of proteins structurally related to angiopoietins, which are growth factors binding the receptor tyrosine kinase Tie2 on endothelial cells and regulating vasculogenesis, vessel homeostasis, and vascular remodeling [107]. Unlike angiopoietins, however, ANGPTLs do not bind to either the Tie2 receptor, or the related protein Tie1, and are therefore considered orphan ligands. ANGPTL4 is considered a member of a new class of proteins named matricellular proteins, which are nonstructural glycoproteins secreted by cancer cells and neighboring stromal cells into the TME, where they associate with the ECM. ANGPTL4 was discovered independently by three groups in the year 2000 as a fasting-induced factor, prevalently expressed in the liver and in the adipose tissue [119]. We now know that ANGPTL4 is a multifaceted protein involved in several metabolic and non-metabolic conditions, in both physiological and pathological situations, including angiogenesis, vascular permeability, tumorigenesis, lipid metabolism, glucose homeostasis, wound healing, and inflammation, among others.

A C-terminal circulating ANGPTL4 fragment (cANGPTL4) binds to ECM proteins and integrins, and this was originally shown to facilitate wound healing [75, 94]. Later, it was found that the binding of cANGPTL4 to integrins β1 and β5 and their subsequent activation regulates cell migration via the focal adhesion kinase (FAK)/p21-activated kinase (PAK)–signaling cascade [25]. Recently, it was demonstrated that cANGPTL4, via the activation of integrin α5β1, increases vascular leakiness by binding to VE-cadherin and claudin-5 and disrupting their intercellular clusters [97]. Furthermore, cANGPTL4 can also associate with specific ECM proteins and delay their proteolytic degradation by MMPs [97]. Thus, ANGPTL4 expression disrupts vascular endothelial tight junctions, augments vessel permeability, and alters trans-endothelial barriers [180], ultimately facilitating tumor cell motility and the formation of metastases. Accordingly, ANGPTL4 was reported to promote venous invasion and distant spread in colorectal and renal cell cancer, as well as in gastric and breast cancer [262]. However, not in every tumor type, ANGPTL4 promotes tumor progression. Indeed, ANGPTL4 was found to prevent lung carcinoma and melanoma metastases via the inhibition of vascular permeability, tumor cell motility, and invasiveness [70]. These contradicting findings may be caused by the different functions of the cleaved forms of ANGPTL4 (N‐or C‐terminus), and highlight the dual role of ANGPTL4 in tumorigenesis.

In HCC and in chronic hepatitis patients, the amount of circulating ANGPTL4 at both mRNA and protein levels is significantly elevated when compared to control individuals [63]. Similarly, in patients with alcoholic liver cirrhosis, serum levels of ANGPTL4 are increased *versus* healthy controls [192]. While these data are suggestive of an oncogenic role of ANGPTL4, other studies reported that both the levels of *ANGPT4* mRNA and the copy number of the gene are lower in HCC samples than in non-tumor tissues of the same patients. A possible mechanism for *ANGPTL4* downregulation in tumors is increased methylation at CpG sites located in the gene promoter [173]. Lower expression levels of *ANGPTL4* mRNA are significantly associated with advanced tumor stage, poor differentiation, tumor recurrence, and decreased post-operative overall and disease-free survival of HCC patients, thereby pointing to a tumor suppressive role of ANGPTL4 [173]. Studies in animal models helped elucidate the role of this protein in liver tumorigenesis. Indeed, the injection of ANGPTL4-overexpressing adenoviral vectors via the portal vein in mice bearing orthotopic liver cancer xenografts resulted in the suppression of both tumor growth and metastasis formation [173]. These findings further supported the inhibitory role of ANGPTL4 in HCC development.

Mutation of the KRAS oncogene is a driver event in PDAC initiation and lineage tracing studies in mice showed that introducing a KRAS^G12D^ activating mutation in acinar/centroacinar cells promotes their differentiation in ductal-like cells and the formation of ADM/PanIN lesions [80]. It was later found that Angptl4 overexpression in these mice increased the number of ADM lesions. Additionally, upregulation of Angptl4 enhanced tumor growth in a xenograft model of PDAC (Panc-1) cells [252]. These findings point to a role for ANGPTL4 in promoting the initiation and progression of pancreatic tumorigenesis.

In patients, four molecularly-defined PDAC subtypes have been identified by integrated genomic analyses, one of them being the squamous subtype associated with mutations in TP53 and KDM6A [14]. *ANGPTL4* expression was the highest in tumors belonging to this subtype, which is associated with a particularly poor prognosis. In an independent study, transcriptome analysis showed that low expression levels of the *ANGPTL4* gene are associated with longer post-operative survival of the patients [125]. Conversely, high *ANGPTL4* expression was observed in patients with shorter survival, as well as in PDAC cell lines resistant to gemcitabine, the standard first-line treatment for advanced or metastatic PDAC [125]. Functionally, knockdown of *ANGPTL4* in a gemcitabine-resistant PDAC line (Panc-1 cells) led to a significant reduction in cell proliferation [125]. Based on these results, ANGPTL4 emerges as a promising candidate target for tumors resistant to gemcitabine, and a potential marker for patients’ stratification in view of this treatment strategy.

Overexpression of *ANGPTL4* is associated with lower disease‐free survival in young breast cancer patients [111]. Moreover, in circulating tumor cells from women with breast cancer, *ANGPTL4* copy number gain was found to be part of a signature of tumor aggressiveness and increased metastatic potential [116].

Triple-negative breast cancer (TNBC) is an aggressive subtype having a dismal prognosis due to its propensity to metastasize to the brain or liver [69]. ANGPTL4 is upregulated in primary tumors, in serum, and in metastases of TNBC patients [26, 96, 97, 161, 180, 216, 256]. Ectopic ANGPTL4 overexpression in a TNBC cell line promoted the formation of 3D mammosphere cultures in vitro and led to the development of larger primary tumors, and to more liver and brain metastases in xenograft models in vivo when compared to cells with endogenous ANGPTL4 levels [220]*.*

Mechanistically, it was shown that soluble ANGPTL4 secreted by metastatic TNBC cells disrupts the integrity of endothelial cell junctions in capillaries within the lungs and brain, thereby allowing tumor cells to access and seed in the respective parenchyma [26, 76, 180].

In conclusion, VEGF signaling promotes disease progression and aggressive behavior in HCC and breast cancer, and consequently, its inhibition has significant anti-tumor effects. In contrast, it does not seem to play a role in PDAC, and this is compatible with PDAC being intrinsically a hypovascular cancer. ANGPTL4 plays a tumor suppressive role in HCC, whereas in PDAC and breast cancer it promotes aggressiveness thanks to its effects on endothelial integrity and cellular migration. The different properties of full-length and truncated ANGPTL4 variants and their tissue distribution likely explain the different phenotypes associated with its upregulation in tumors. Targeting ANGPTL4 is considered a promising strategy to reduce tumor growth in PDAC and metastases formation in breast cancer (Fig. 2).

### Angiogenesis in healthy and pathological adipose tissue

The adipose tissue is a highly vascularized tissue. In addition to nutrients and oxygen, the blood vessels transport growth factors, cytokines, and hormones that are required for adipocyte function, growth, and survival [35]. Furthermore, the vasculature regulates the transport of adipokines from the adipose tissue to other organs, thereby mediating the endocrine role of this tissue in controlling systemic energy balance and metabolic activity in peripheral tissues.

The adipose tissue is the most plastic tissue in multicellular organisms, as it constantly undergoes dynamic remodeling to adapt to ever-changing nutritional conditions. This plasticity goes hand-in-hand with a remarkable angiogenic capacity. While during weight gain, expansion of the adipose tissue is accompanied by an increase in vascularization via angiogenesis, weight loss is associated with the regression of blood vessels. In the adipose tissue, the vasculature is also critical for the effective local removal of free fatty acids (FFAs) during fasting, making angiogenesis a rate-limiting step for fat tissue expansion [230] (Fig. 2).

In obesity, substantial changes in adipose tissue structure are observed, which are not limited to adipogenesis, but also involve angiogenesis. Higher nutrient availability causes an increase in the number (hyperplasia) and size (hypertrophy) of the adipocytes, which reduces oxygen availability and induces a mild hypoxic state. To reduce hypoxia, adipose tissue promotes angiogenesis. HIF1α was found to be increased in the adipose tissue of obese patients, and its expression was reduced upon weight loss following bypass surgery [32]. As for tumor tissues, upregulation of HIF1α activates the transcription of the pro-angiogenic factors *ANGPTL4* and *VEGFA* [34, 48, 223].

#### Angiogenesis mediators in obesity: potential therapeutic targets.

A functional vasculature is essential for both physiological adipose tissue expansion and for its expansion in obesity. Thus, not surprisingly, circulating VEGFA levels are elevated in overweight and obese individuals [162] and have been found positively correlated to BMI in healthy male subjects [145].

Several studies in animal models have shown that disruption of angiogenesis prevents the onset of obesity [50]. VEGFA mediates most of the pro-angiogenic activity in adipose tissue by binding to VEGFR1 and VEGFR2 on endothelial cells, and leading to proliferation, mitogenesis, and growth factor secretion. Using doxycycline-inducible transgenic mice overexpressing VEGFA specifically in adipose tissue, it was demonstrated that angiogenesis expedites the expansion of healthy fat pads following a high-fat diet (HFD) regimen [230]. Moreover, these mice had improved insulin sensitivity and increased energy expenditure on HFD, as well as a lack of fibrosis and inflammation in adipose tissue. This was likely due to VEGFA-mediated new vessel formation and the related decrease in hypoxia. These data suggest that, in mice, promoting angiogenesis in the early stages of obesity may be beneficial, thereby opening new avenues for the potential use of manipulating angiogenesis via VEGFA administration in obese patients. It was recently reported that high fat-high sucrose feeding in mice increases HIF1α-mediated VEGF expression in hypothalamic astrocytes, and this promotes microvascular remodeling and could then lead to obesity-related hypertension [79].

Originally identified as a fasting-induced factor, ANGPTL4 was later shown to regulate lipid metabolism by inhibiting the lipoprotein lipase (LPL) enzyme. ANGPTL4 binds to and inhibits extracellular LPL activity, and stimulates the lipolysis of triacylglycerol stored by adipocytes. The binding to LPL occurs via the N-terminal nANGPTL4 fragment, the fragment responsible for the oligomeric assembly of the protein. Mutations that prevent ANGPTL4 oligomerization severely compromise its ability to inhibit LPL [73]. LPL is an enzyme that hydrolyzes triglycerides embedded in lipoproteins, such as very low-density lipoproteins (VLDL), and in chylomicrons travelling through the bloodstream. The normal function and activity of LPL are essential for maintaining a well-balanced metabolism of plasma triglycerides, and as such are tightly regulated, mostly at post-translational level. Indeed, while the mRNA levels of *LPL* in adipose tissue are similar in both the fed and fasted states [118], the rate of LPL degradation within the Golgi/postGolgi secretory compartment increases during fasting. Moreover, in the fasted state, there is a conversion of active LPL into its inactive form [21], and this shift is caused by the induction of *ANGPTL4* expression in adipose tissue [229].

While the CCD fragment is responsible for the inhibition of LPL activity, the purified FLD fragment of Angptl4 stimulates lipolysis in mouse primary adipocytes in vitro [159]. Moreover, adenovirus-mediated overexpression of the FLD fragment (Ad-FLD) in mice, which increases the circulating FLD levels, was found to promote adipose tissue lipolysis in vivo, as well as to reduce diet-induced obesity. Noteworthy, decreased adiposity in Ad-FLD mice was associated with increased oxygen consumption, fat utilization, and expression of thermogenic genes in subcutaneous adipose tissue [159]. These studies revealed that the FLD fragment, when separated from the CCD LPL-inhibitory fragment, displays lipolytic and thermogenic properties, which could be relevant in the context of the identification of novel treatments for obesity and diabetes.

In humans, a large population study demonstrated a positive correlation between plasma ANGPTL4 concentrations and fasting plasma glucose [117]. Additional human genetics studies further supported the role of ANGPTL4 in regulating plasma lipid levels by showing that carriers of loss-of-function variants in ANGPTL4 (the most common being E40K) show lower plasma TG levels than non-carriers [52, 203]. The E40K variant, which encodes an unstable ANGPTL4 protein [228, 255], is also associated with a significantly reduced risk of coronary artery disease [126, 148, 228, 255].

Interestingly, circulating ANGPTL4 levels were found to be elevated in obese patients with different metabolic phenotypes, i.e., metabolically unhealthy, type 2 diabetes (T2D), and metabolically healthy, and this was associated with endothelial dysfunction, an early abnormality in atherosclerosis [207]. ANGPTL4 levels were especially higher in the group of patients with T2D than in any other group, in agreement with another study showing that the plasma levels of ANGPTL4 are almost two-fold higher in patients with T2D than in nondiabetic controls [253]. Further clues about the role of Angptl4 in metabolism and obesity came from genetically engineered mice with deletion of the gene. *Angptl4*-knockout mice fed a HFD were reported to develop a severe inflammatory phenotype, which led to their premature death and precluded further studies on the role of Angptl4 in diet-induced obesity [138]. However, when unsaturated fatty acids and cholesterol were added to a chow diet, and fructose to the drinking water, *Angptl4*-knockout mice had much better glucose tolerance than wild-type mice, despite being more obese [108]. It should be noted that the phenotype can vary depending on the genetic background of the mice [12]. Studies of mice with the conditional depletion of *Angptl4* in adipose tissue demonstrated that, under conditions of diet-induced obesity, gene deletion reduces circulating triglycerides and improves insulin sensitivity, whereas body weight and glucose homeostasis remained unchanged [7]. Mice with hepatocyte-specific *Angptl4* gene fed a chow diet showed early changes in lipid metabolism, with reduced circulating triglycerides, total cholesterol, and HDL cholesterol [221]. Noteworthy, liver-specific Angptl4-deficient mice fed a HFD for 16 weeks showed decreased body weight, plasma lipids, and liver steatosis, together with improved glucose tolerance and insulin sensitivity [221]. These phenotypes were mediated by an increase in FFA uptake in hepatocytes, which led to their oxidation, and ultimately to ROS production, and AMPK activation [221]. The observation that pharmacologic inhibition of Angptl4 in the liver recapitulates the amelioration of metabolic parameters observed following its genetic ablation (i.e., protection against diet-induced obesity, dyslipidemia, glucose intolerance, and liver damage) highlighted the potential of this factor as a therapeutic target for metabolic diseases.

In conclusion, the inactivation of Angptl4 in mice plays a protective role against the metabolic complications of diet-induced obesity, so that it is currently investigated as a therapeutic strategy for dyslipidemia and to improve glucose intolerance [108].

### Angiogenesis linking obesity and cancer

Epidemiological studies have shown that obesity increases the risk of developing liver cancer [30] because of its role in the progression of NAFLD to cirrhosis [29], the single most potent risk factor for the development of HCC. Given that angiogenesis is associated with the progression from cirrhosis to HCC, it could be hypothesized that increased angiogenesis links obesity to worse outcome in HCC patients. To verify this hypothesis, Siegel and colleagues set out to investigate the amount of microvascular invasion (MVI), a sign of angiogenic activity associated with poorer prognosis, and found that it positively correlated with BMI in HCC patients [219]. In the TME, adipokines secreted by the adipose tissue (e.g., leptin) impinge on the increase of VEGF and sustain neovascularization, further strengthening the importance of angiogenesis in the relationship between obesity and HCC development (Fig. 2).

A similar situation occurs in PDAC, where excess adiposity is causally related to the development and the severity of the disease, and where excess in intrapancreatic fat is an indicator of PDAC [38]. Noteworthy, fatty pancreas and fatty liver are strongly correlated, and both are associated with obesity [241]. In orthotopic PDAC models, HFD resulted in a more aggressive tumor phenotype and this was accompanied by an increase in angiogenesis [71]. Whether the increased expression of pro-angiogenic factors (e.g. VEGFA, ANGPTL4) is involved in mediating tumor progression was not investigated.

Obesity is associated with a higher risk of developing estrogen receptor-positive breast cancer, particularly in postmenopausal women, as well as with a worse clinical outcome for women of all ages. Several studies have demonstrated that the secretion of inflammatory cytokines, growth factors, and fatty acids by the adipose tissue in the TME exerts protumorigenic effects, which ultimately promote breast cancer progression (Fig. 2). In mouse models of breast cancer,  the adipose tissue sustains the growth of the new vasculature and supports the development and progression of the tumors via the secretion of VEGFA and other angiogenic factors [40]. Blücher and colleagues analyzed the effects of factors secreted by adipose cells of obese mice or obese patients on several oncogenic features of breast cancer cells [25]. They found that these factors activate the expression of genes involved in inflammation and lipid metabolism, including *ANGPTL4*, and this was associated with increased tumor cell proliferation and invasion (Fig. 2). In mice, obesity-related inflammation causes an increase in IL-1β production, which then promotes angiogenesis and tumor progression by upregulating *Angptl4*, thereby setting the rationale for future exploitation of this factor as a potential therapeutic target for obese breast cancer patients [129]. To the local pro-tumorigenic effects of the adipose tissue are added systemic endocrine changes, such as increased estrogens, insulin, and leptin levels, that may promote breast cancer development and progression. Leptin signaling, known to be associated with breast cancer aggressiveness and worse prognosis [158, 200], activates transcription factors that upregulate VEGF/VEGFR2 to promote angiogenesis [41].

The response to therapy is affected by body weight. For instance, it has been shown that syngeneic murine breast cancer models fed a HFD *versus* a low-fat diet, as well as obese breast cancer patients, are resistant to anti-VEGF therapy [101]. This results from increased production of IL-6 and FGF2 by adipocytes and myeloid cells, given that blocking IL-6 or inhibiting FGF in preclinical models of primary and metastatic breast cancer restores the response to anti-VEGF therapy [101]. Whether obesity also affects the response of HCC patients to the anti-angiogenic drug sorafenib is still unclear. Indeed, while a small retrospective study reported that HCC patients with high visceral fat display both shorter overall survival (OS) and primary resistance to sorafenib [171], another one failed to confirm a negative prognostic value of individual components of the metabolic syndrome (including obesity) on OS [133]. Despite these inconclusive findings, there is a need to develop specific therapies for better treating obese cancer patients.

Based on animal studies, administration of VEGF in the early stages of obesity could be beneficial in reducing the tissue-related changes associated with this condition, and could therefore prevent the development of tumors which are associated with obesity. However, once tumors occur, VEGF signaling should be blocked to prevent angiogenesis and further tumor growth and spread. Similarly, blocking Angptl4 is expected to lead to tumor inhibition in the context of obesity, as it reduces the angiogenic potential of the tumors (Fig. 2).

## Adrenergic signaling linking obesity and cancer

### Adrenergic signaling mediates our body’s fight-or-flight response

Activation of adrenergic receptors through the catecholamines norepinephrine or epinephrine triggers our body’s fight-or-flight response, the reaction to immediate stress. It also activates multiple downstream cascades involved in metabolism, proliferation, inflammation, DNA damage response, and other processes relevant for both cancer and obesity. Adrenergic receptors are G-protein-coupled receptors. In humans, there are three classes of adrenergic receptors (α1, α2, β) and 9 subtypes in total. The different adrenergic receptors are expressed in a tissue-specific manner and signal through distinct biochemical pathways [49]. Depending on the G-protein to which they are coupled, they can activate multiple pathways, most prominently the cAMP signaling cascade, activation of which leads to adenylate cyclase activation and cyclic 3′–5′ adenosine monophosphate (cAMP) production. cAMP then activates the effector proteins protein kinase A (PKA) or exchange protein activated by adenylyl cyclase (EPAC), ultimately resulting in transcriptional regulation of a multitude of genes regulating cell fate [43].

### Increased local and systemic adrenergic signaling contributes to cancer development and progression

Adrenergic signaling is an important regulator of different cellular processes linked to cancer development and progression, such as angiogenesis, cell motility, and inflammation. Adrenergic receptors are expressed not only on tumor cells [195] but also on cells of the TME, such as immune cells or vascular cells [128, 257]. Catecholamines can act both locally and systemically, and both pathways seem to play a role in cancer. Several studies argue that the local adrenergic signaling is of high importance for cancer progression: In mouse models of prostate cancer, catecholamines derived from adrenergic nerves activated β-adrenergic receptor signaling in endothelial cells, which led to an angiogenic switch promoting tumor growth [257]. In patients with oral cancer, nerve density was associated with p53 status, which correlated with poor clinical outcomes [3]. Thus, the tumor-nerve crosstalk seemed to play an important role in adrenergic signaling in cancer. Catecholamine levels in ovarian carcinomas were higher in tumor tissue than in blood, and intra-tumor but not circulating norepinephrine levels correlated with tumor grade and stage [151], again arguing for the importance of local adrenergic signaling in cancer.

In addition to local signaling, there is also compelling evidence for the importance of systemic catecholamine signaling in cancer: Increased cancer cell proliferation upon treatment with the synthetic β-adrenergic receptor agonist isoproterenol, which could be prevented through blockage of β-adrenergic signaling [211], first gave rise to the idea that catecholamines might play a role in cancer growth. Interest in adrenergic signaling in cancer was further sparked by the observation that chronic stress, which is linked to the activation of adrenergic signaling, was associated with a higher cancer incidence. Social isolation, which is an enormous stressor in rats, vastly increased cancer occurrence and malignancy such that mammary tumor burden in isolated rats was increased to > 80 times that of age-matched control animals housed in groups [91]. Likewise, although much less drastic, chronic behavioral stress resulted in greater tumor burden and more invasive cancer cell growth in mice orthotopically implanted with ovarian carcinoma cells [235]. This was linked to over-activation of the β2-adrenergic receptor (ADRB2)-cAMP-PKA pathway and increased angiogenesis in the stressed situation. There is now a large body of data from animals showing that adrenergic signaling is involved in tumor growth, metastasis, and overall mortality associated with multiple cancers, such as melanoma [17], colorectal cancer [141], breast cancer [222], and many more. In line with the rodent data, an early cohort study investigating over 10,000 women found significantly increased breast cancer risk in patients who had experienced a stressful life event, such as a divorce or death of a close family member [139]. The risk was also increased by stressful events among twin pairs, although the cohort size was small in this case. More recently, a large meta-analysis showed a significant association between work stress and the risk of different cancers, including those of the colon, lung, and esophagus [254].

### Beta-blockers in cancer treatment

Beta-blockers are antagonists of beta-adrenergic receptor signaling which belong to a class of drugs used primarily for the treatment of cardiovascular diseases. There are both nonselective agents and agents selective to certain β-adrenergic receptors. Common examples include propranolol, carvedilol (nonselective), atenolol, and metoprolol (β_1_-selective). Initially shown and later confirmed many times in preclinical studies [157, 235, 247] antagonism of β-adrenergic signaling inhibited multiple pathways involved in cancer progression and metastasis. Given the large body of evidence linking adrenergic signaling and cancer from both preclinical and clinical studies, the use of beta-blockers in cancer treatment seemed promising. Evidence of a positive effect of beta-blockers on cancer outcomes is most compelling for breast cancer: A series of population-based observational studies demonstrated significantly reduced breast cancer progression and mortality in patients treated with the nonselective beta-blocker propranolol [16]. Herein, the authors confirmed observations of the positive effect of this beta-blocker in preclinical trials. In patients with triple-negative breast cancer, beta-blocker use was linked to improved relapse-free survival [146]. Beta-blocker therapy was also beneficial in the context of metastasis formation, as a significantly lower rate of distant metastasis, reduced cancer recurrence, and improved cancer-specific survival were observed upon beta-blocker therapy in breast cancer [191]. This study concluded that cancer patients receiving beta-blockers for hypertension had improved overall outcomes, but larger studies are needed to make definitive conclusions. In addition to direct effects of tumor growth and/or metastasis formation, there seem to also be additional advantages of beta-blockers, for instance when considering chemotherapy: Many traditional chemotherapies induce cardiotoxic side effects, such as reported for cisplatin (arrhythmias, angina, myocarditis, acute myocardial infarction, and chronic heart failure, and more [187]. Due to their beneficial function on the heart, beta-blockers may be useful to improve cardiac health during chemotherapy. Indeed, beta-blocker treatment reduced the risk of new heart failure diagnosis in breast cancer patients undergoing chemotherapy [81].

Beta-blockers have also been discussed in the context of pancreatic cancer. Stress-induced neural activation increased tumor growth and cancer cell migration in a mouse model of pancreatic cancer, which was blocked by pharmacological β-blockade [121]. Herein, optical imaging was used to track pancreatic cancer cells in vivo. Pharmacological activation of beta-adrenergic signaling induced increased primary tumor growth as well as cancer cell migration to the same extent as stress through repeated daily restraint, underlining the relevance of this pathway to stress-induced aggravation of cancer progression. A recent study showed that catecholamines promoted the development of PDAC through ADRB2 in a feedforward loop involving neurotrophins, sympathetic innervation, and local norepinephrine [198]. In turn, ADRB2 blockade increased the survival of mice prone to pancreatic cancer. This effect was mediated primarily through local catecholamine action, as indicated by the protection from cancer growth by surgical sympathectomy [198]. In a syngeneic murine model of pancreatic cancer, animals with high circulating catecholamines, as in chronic stress, also had larger tumors and reduced survival [185]. Propranolol treatment significantly improved both tumor growth and survival of the animals. In patients with PDAC, beta-blocker treatment was associated with significantly reduced cancer-specific mortality—an effect that was particularly pronounced in patients with localized disease at diagnosis [239]. Likewise, the beta-blocker propranolol reduced mortality in patients suffering from hepatocellular carcinoma [39, 238]. Despite this compelling evidence of a beneficial role of beta-blockers in cancer, other studies fail to observe beneficial effects: a recent large meta-analysis including > 18,000 patients has not shown any advantages of beta-blockers regarding overall deaths, cancer-specific deaths, and recurrences in breast cancer [123, 124]. This was also true for other cancer types [263], and there now is considerable debate whether or not the stress-cancer link is true, as there are also several large studies demonstrating no increased cancer incidence in different stress conditions [88, 208]. Overall, there is no clear and uniform picture, both regarding the stress-and-cancer link and regarding the benefit of beta-blockers in cancer treatment—although multiple clinical studies are currently ongoing. It should be noted that none of these studies has systematically addressed obesity. The link between adrenergic signaling and cancer may be influenced by the interaction with the metabolic action of adrenergic receptors as discussed below.

### Beta-blockers in the treatment of cancer cachexia

Beta-blockers have not only been assessed for the treatment of cardiovascular disease and tumors, but also for cancer-associated metabolic dysfunction. Cancer is frequently associated with cancer cachexia, a wasting disorder caused by various tumor entities, that is estimated to cause every third cancer-related death and strongly impairs quality of life, and negatively affects outcomes of anti-cancer therapies [65]. Cachexia leads to the involuntary, progressive loss of adipose tissue and muscle mass and cardiac dysfunction [166, 201, 226]. Being a systemic metabolic disease, weight loss in cachexia is associated with increased adrenergic signaling, which increases energy expenditure, and can be blocked by β_3_-adrenergic receptor antagonists in mouse models [110, 188]. Beta-adrenergic signaling is a key activator of adipocyte lipolysis and regulation of which contributes not only to adipose tissue size but also overall body weight and metabolic health [202]. Over activation of adipocyte lipolysis is a hallmark of cancer cachexia, and blockage of this process counteracts cancer-induced wasting [51, 201]. Prominently, ADRB1 was shown to be over-expressed in the adipose tissue of patients with cachexia, correlating with elevated expression of hormone-sensitive lipase, a rate-limiting enzyme in lipolysis, and the lipolytic rate [33]. Given the relevance of cardiac function for survival in cancer patients, the finding that beta-blockers also reduced the negative consequences of cancer on the heart and reduced mortality in rodents [226] proposes that beta-blockers may also be useful treatment options in cancer-associated cachexia. In addition, cancer cachexia has been associated with increased intratumoral TGFβ signaling and tumor fibrosis [140]. Notably, cancer cachexia represents a systemic metabolic syndrome associated with malignancy, which in turn might contribute to tumor aggressiveness through the outlined processes, namely induced tumor fibrosis and angiogenesis.

### Adrenergic signaling affects cancer progression through multiple mechanisms

There are multiple ways through which adrenergic signaling may contribute to cancer growth and progression. Catecholamines directly activate proliferation, and beta-blockers may function through inhibiting this pathway as shown by Montoya et al. [164]. In this study, the authors demonstrate overexpression of β_1_- and β_3_-adrenergic receptors in breast cancer and reduced tumor proliferation in primary breast cancer cells and a panel of mammary epithelial cell lines. Mechanistically, this was due to decreased phosphorylation of the mitogenic signaling regulators p44/42 MAPK, p38 MAPK, JNK, and CREB, and increased phosphorylation of the cell survival/apoptosis regulators AKT, p53, and GSK3β. This direct proliferative effect—and blockage through beta-blockers—was also observed in multiple other cancer cell lines [42, 142, 211]. Beta-blockers were not only able to block the increased proliferation of cancer cells induced by beta-adrenergic receptor agonists but also reduced baseline proliferation. In addition to the above-mentioned p44/42 MAPK and AKT, also Bcl-2, cyclin D1, and cyclin E were suppressed by beta-blockers, causing G1/S-phase arrest, and ultimately caspase activation and apoptosis, as shown by [259]. Interestingly, however, there are also reports on reduced cancer cell proliferation upon β-adrenergic signaling [103]. In addition to cell lines, increased tumor cell proliferation mediated by catecholamines was also observed in vivo in mice [185]. However, the same study also noted an even more pronounced effect of catecholamines on cell migration, which was inhibited by beta-blockers. In this study, chronic stress-induced higher expression of VEGF, increased micro vessel density, and increased expression of MMP9. Augmented migration of human prostate cancer cells upon norepinephrine treatment was also seen in a mouse xenograft model [15]. Herein, norepinephrine directly activated the migration of cancer cells. Knockdown of β2-adrenergic receptor in breast cancer cells reduced tumor cell invasive capacity and abolished stress-induced metastasis, whereas overexpression of the receptor in low metastatic cells induced an invasive phenotype [37]. One potential explanation for this increased metastasizing behavior may be the elevated expression of matrix metalloproteases, most importantly MMP2 and MMP9, which has been shown in multiple cancer cell lines upon β-adrenergic signaling, and importantly improved by propranolol treatment [15, 82]. When treated with norepinephrine, pancreatic cancer cells secreted significant amounts of these MMPs. Being important molecules involved in the degradation of ECM components, MMPs are critical to both metastasis and angiogenesis processes [194], as well as to liver fibrosis [134]. Likewise, increased VEGF secretion upon NE treatment facilitated angiogenesis and metastasis formation, as outlined in chapter 2. Angiogenic processes were vital for mediating the effects of stress on tumor growth in a mouse model of ovarian cancer [235], which again showed an increased expression of VEGF, MMP2, and MMP9. MMPs are also associated with EMT, a crucial factor determining cancer cell invasion and metastasis. In addition to inducing MMP expression and secretion, catecholamines activate other processes typical for EMT, as they induce morphological characteristics of EMT as well as increased vimentin and decreased E-cadherin expression [217]. Mechanistically, this was driven through a β_2_-adrenergic receptor—HIF1α—Snail axis, as HIF1α knockdown prevented EMT in cancer cells.

In addition to these direct effects, a possible explanation for the increased migratory capacity upon adrenergic signaling might be the interconnection with the immune system [222]. Catecholamines induced the expression and secretion of inflammatory mediators, such as interleukin 6 or 8 (IL6, IL8) from human cancer cells [165]. Here, the main role of IL6 was to participate in the activation of stromal fibroblasts towards a myofibroblastic phenotype, supporting metastasis. In addition, inflammatory mediators have vital roles in orchestrating the various cell types and responses of the immune system. β-adrenergic signaling increased the infiltration of CD11b + F4/80 + macrophages into the primary tumor and thereby induced a prometastatic gene expression signature accompanied by indications of M2 (non-tumoricidal) macrophage differentiation [222]. While stress-induced neuroendocrine activation had a negligible effect on the growth of the primary tumor in this study, it led to a 30-fold increase in metastasis to distant tissues including the lymph nodes and lung, underlining the close interconnection of these two processes. In addition, beta-adrenergic signaling enhanced the production of prostaglandins, specifically prostaglandin E2 (PGE2), through a mechanism involving nuclear factor kappa b (NFκB) signaling, which proved essential for mediating the stress-induced increase in tumor growth and metastasis formation in mice [170]. Increased expression levels of key proteins involved in PGE2 production were also observed in human ovarian cancer samples and were linked to reduced survival of these patients. Consequently, there have already been small trials investigating the effects or propranolol and a COX2 inhibitor, etodolac—reducing PGE2 synthesis—in cancer patients, which showed promising first results in terms of metastatic potential and inflammatory signatures [86, 215]. Lastly, beta-adrenergic signaling was also shown to impair CD8 + T cell activation by suppressing the metabolic switch leading to activation of effector function, thereby limiting anti-tumor T cell action and efficacy of immunotherapies [175].

Overall, there is a very strong link between adrenergic signaling and cancer, and multiple pathways important for cancer development and progression are affected by stress signaling which also interconnect with angiogenic and ECM pathways as outlined above. Despite this, clinical evidence for the effectiveness of beta-blockers is not unequivocal, which may be due to the so far neglected connection to metabolic pathways as discussed below.

### Adrenergic signaling and obesity

Dysfunction of the adrenergic system has been proposed in obesity, as the sympathetic nervous system influences both energy intake and energy expenditure. Beta-adrenergic receptors regulate on the one hand metabolic rate and substrate utilization, and on the other hand contribute to the regulation of the function of multiple tissues important for metabolic control [236]: Adrenergic signaling regulates carbohydrate metabolism in liver and skeletal muscle, insulin and glucagon secretion from the pancreas (thereby influencing systemic glucose homeostasis), and lipid metabolism in the adipose tissue. Low sympathetic nervous activity has been suggested as potential mechanism of weight gain in humans [225]. Adipose tissue adrenergic signaling seems key to most effects linking adrenergic signaling and body weight regulation. Beta-adrenergic receptor expression is strongly reduced in adipose tissues of several different mouse models of obesity, making impaired expression a general feature of both genetic and diet-induced obesity. Comparing different models, the degree of obesity was correlated with the extent of loss of β1- and β3-adrenergic receptor expression in the adipose tissue [45]. Likewise, it was noted that factors that downregulate β-adrenergic receptor expression induce both obesity and associated metabolic dysfunction due to impaired lipid mobilization [202]. On the other hand, chronic activation of β-adrenergic signaling by CL316,243 treatment prevented the reduction in β1- and β3-adrenergic receptor expression in the adipose depots, and prevented diet-induced obesity [44]. Catecholamines are key regulators of lipolysis in the adipose tissue, and β-adrenergic signaling also activates thermogenesis, leading to energy expenditure through heat. This not only renders adipose tissue adrenergic signaling important for cancer-associated cachexia as mentioned previously but also makes adipose tissue β-ARs attractive drug targets to counteract obesity [6]. Obesity-associated catecholamine resistance of adipocytes is well established in both rodent models and patients [199]. A recent report has highlighted a novel regulatory pathway involved in β3-adrenergic receptor desensitization in obese adipocytes which involves the pseudokinase Tribbles 1[179], the EPAC pathway, and the transcription factor CEBPα [240]. Previously, inflammatory processes associated with obesity have been shown to mediate obesity-induced catecholamine resistance in adipose tissue [167]. In that regard, inflammatory macrophages have been shown to reduce adrenergic output by degrading catecholamines in the adipose tissue, thereby limiting catecholamine bioavailability [31, 190]. This highlights an important point: while catecholamines are either locally reduced or less functional in the adipose tissue of the obese—a mechanism that has been pursued intensively as a drug target in recent years—obesity is actually associated with increased circulating catecholamines and elevated sympathetic tone [237]. Multiple factors may induce increased sympathetic activity in obesity: overfeeding-induced disruption of hypothalamic insulin-signaling, hyperinsulinemia, hyperleptinemia, and increased release of nonesterified fatty acids from adipose tissue [236]. Activation of the sympathetic nervous system may also explain the markedly increased risk of hypertension and cardiovascular disease in obesity [113], although multiple additional factors such as adipokines or the renin–angiotensin–aldosterone system contribute to mediating blood pressure and cardiac outcomes. Plasma norepinephrine levels are significantly higher in obese patients, and weight loss in obese subjects is correlated with reductions in plasma norepinephrine [237]. This elevated sympathetic activation is further highlighted by the study by Grassi et al. demonstrating that muscle sympathetic nerve activity was much higher in obese compared to healthy subjects [78]. A recent meta-analysis combining data from 45 studies has shown that muscle sympathetic nerve traffic, a sign of sympathetic activation, was higher in overweight subjects compared to subjects with normal weight, and higher still in obese subjects. Indeed, muscle sympathetic nerve traffic was directly related to body mass index and waist-to-hip ratio [77].

### Elevated adrenergic signaling may link obesity to enhanced cancer progression

**Fig. 3:**
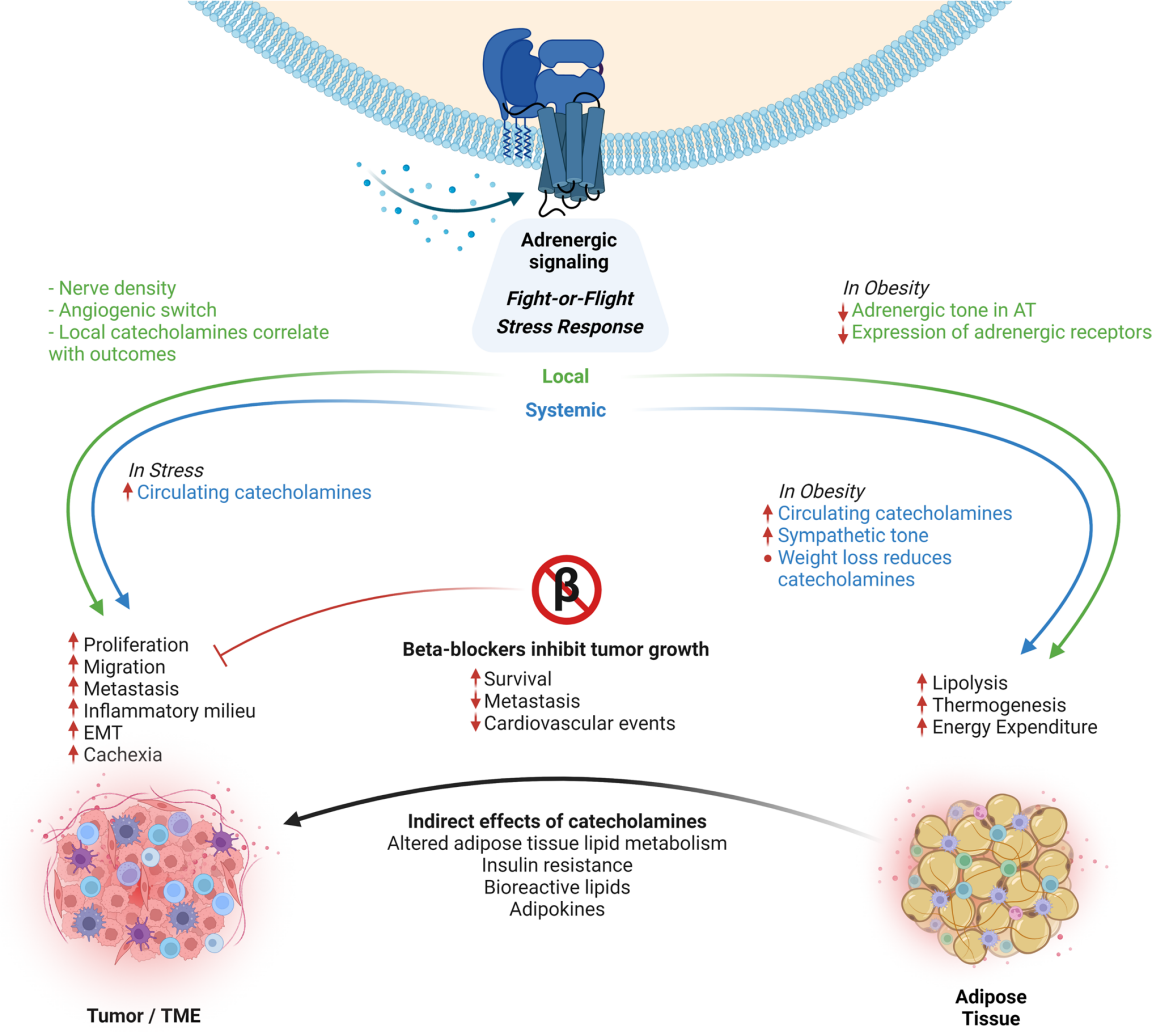
Overview of adrenergic signaling in cancer and obesity. Adrenergic signaling activates lipolysis, thermogenesis, and energy expenditure in the adipose tissue. Within tumor and tumor microenvironment, adrenergic signaling activates different pro-tumorigenic processes including tumor cell proliferation, angiogenic switch, and epithelial-mesenchymal transition (EMT). Obesity is associated with elevated circulating catecholamines, which can be reduced upon weight loss, but adrenergic signaling is dampened locally within the adipose tissue, contributing to reduced energy expenditure and impaired adipose tissue function. This affects tumors through indirect catecholamine-mediated effects including bioreactive lipids and adipokines. Elevated circulating catecholamines are also present in stress. Many direct tumor-promoting processes, as well as tumor-associated complications (cardiovascular events, cachexia), can be counteracted by beta-blockers. The lack of associations between stress or beta-blockers and cancer outcomes in some studies may result from variabilities in adrenergic signaling due to obesity

The pronounced elevation of the sympathetic nervous system in obesity may contribute to enhanced cancer progression in obese patients (Fig. 3). A polymorphism in the β_3_-adrenergic receptor gene was associated with obesity and metabolic syndrome and was also highly significantly enriched in patients with endometrial cancer [11]. Likewise, an association between the risk of breast cancer and polymorphisms in ADRB2 and ADRB3 was described [98]. Herein, the authors suggested that variations in adrenergic receptors linked to obesity may also be crucial risk factors for postmenopausal breast cancer. In addition to the direct effects of elevated sympathetic activation on cancer cells through the molecular mechanisms described above, it is conceivable that catecholamines also indirectly advance tumor growth, for instance by altering the lipid metabolism in the adipose tissue. There may also be an interesting connection with leptin: increased leptin secretion from adipose tissue and elevated circulating leptin are seen in obesity, and indeed an association between leptin and norepinephrine exists, for instance during a glucose tolerance test [46]. This association may reflect the lack of leptin suppression by catecholamines in obesity, which occurs in the dependence of β_1_- and β_2_-adrenergic receptors in healthy human adipocytes [212]. In addition, sympathetic nervous system overactivation is associated with hypertension, which itself is a risk factor for the increased incidence of developing certain cancers and increased cancer-related mortality [163]. Over activation of beta-adrenergic signaling, as seen in obesity, may additionally potentiate insulin resistance by directly interfering with glucose uptake and insulin signaling as recently shown in response to acute stress, and this mechanism might also occur in obesity [196].

There are also important implications for cancer-associated complications such as cachexia. As cachexia is classically associated with increased activation of adrenergic receptors, for instance in the adipose tissue [188], a so-far unknown connection with obesity may exist. Lastly, it should be noted that adrenergic signaling is also activated in exercise, which has known anti-tumor effects, and that blocking adrenergic signaling upon exercise prevented the anti-tumor action of exercise in mouse models of cancer through interaction with the immune system [109]. Preclinical studies have shown primarily positive effects of beta-blockers on cancer, whereas this was not always the case in the clinical data. One difference may be weight homogeneity in mouse models, and future studies investigating adrenergic signaling and effects of beta-blockers in cancer should pay closer attention to body mass and body composition. It remains an interesting question if and how we can use our knowledge on obesity for cancer therapy. For instance, as outlined above, macrophages locally degrade catecholamines in obese adipose tissue—could we train them to perform the same action in cancer, thereby reducing local catecholamine bioavailability in the tumor? Many open questions regarding spatio-temporal actions of adrenergic signaling in obesity and cancer still exist, and carefully assessing the metabolic status of patients may aid in understanding the benefit of intervening with adrenergic signaling in a more personalized setting in the future.

## Conclusions

**Fig. 4:**
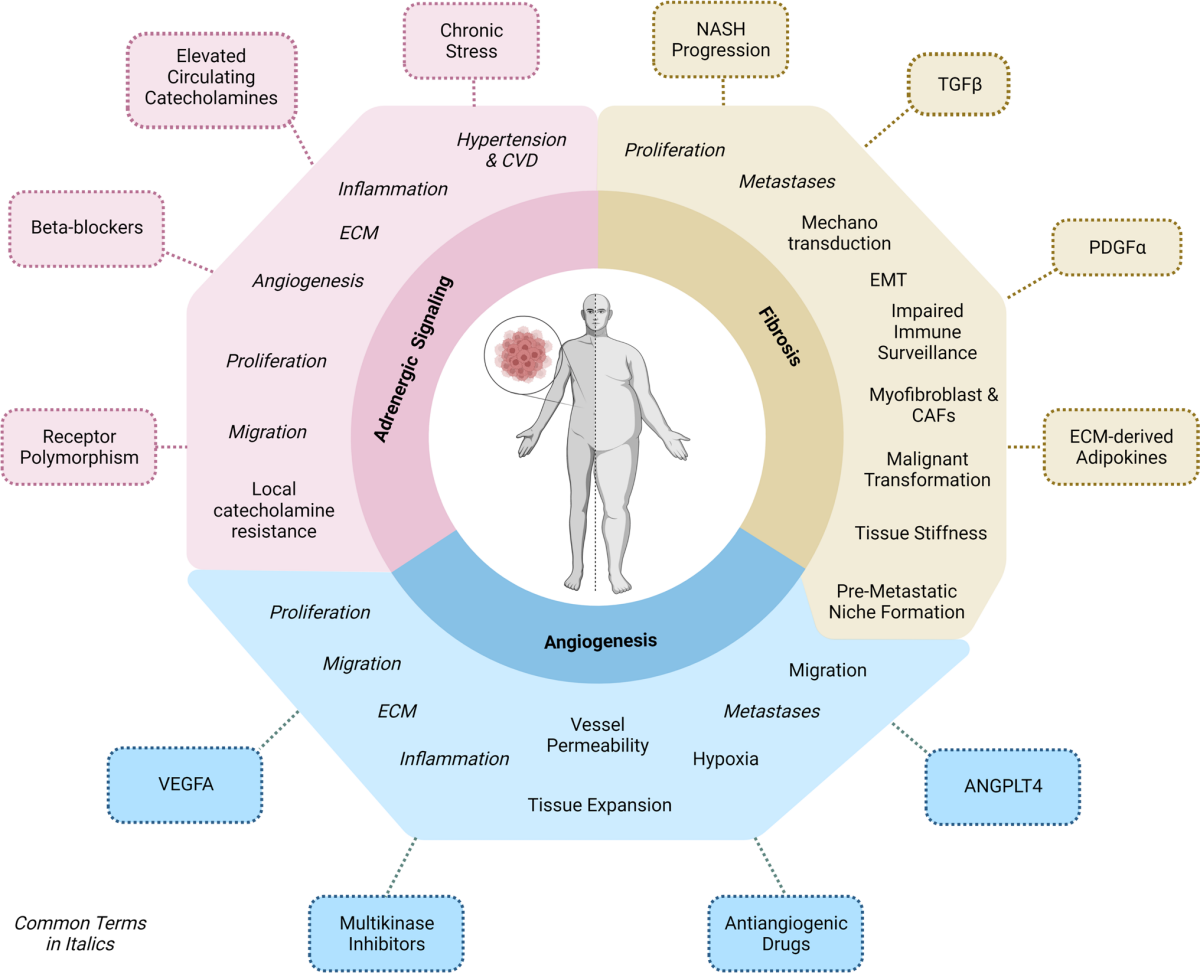
Schematic representation of fibrosis-, angiogenesis- and beta-adrenergic signaling-related processes and factors contributing to the risk connection between obesity and cancer

Treatment options for cancer patients are the same whether they occur in obese or lean individuals, but body weight and composition might affect therapy response and toxicity. Therefore, there is a need to develop more effective and tailored therapies that take into account the patients’ metabolic status. Several processes, including fibrosis, angiogenesis, and adrenergic signaling, are located at the crossroad between obesity and cancer (Fig. 4). Molecules involved in these processes represent putative therapeutic targets that may be exploited in view of future clinical applications. Importantly, the role that each molecular mediator plays in the individual cancer types (pro- or anti-oncogenic), as well as in the development of obesity-associated complications, needs to be taken into account when planning a targeting strategy. Considering body weight and body composition in a systematic manner when designing and interpreting cancer data will further increase the impact of these studies.

While obesity represents an undisputed risk factor for cancer development and is associated with more aggressive cancer behavior, weight loss has been correlated to reduced cancer incidence, especially of breast and endometrial cancers in post-menopausal women [184]. Studies in experimental animals back up this finding by showing that caloric restriction decreases tumor formation and slows tumor growth (reviewed in Lv et al. [152]. For instance, caloric restriction reduces sympathetic activity to an extent comparable to beta-blockers in rodents [174]. Thus, weight loss represents a good intervention measure for the prevention of several human cancers, including HCC, PDAC, and breast cancer. This is especially true for interventions leading to durable and significant weight loss such as bariatric surgery. A recent population-based study has shown that bariatric surgery decreases the risk of obesity-associated cancers, but does not change the incidence of cancers not related to obesity [136]. In the future, raising awareness about the connections between metabolic diseases and their long-term consequence on cancer among patients, physicians, and the research community will be critical to prevent the disease or improve treatment outcomes in cancer. In those patients unable to lose weight, exploring the mechanistic links between obesity and cancer progression may provide novel treatment options in the future and potentially improve the current standard of care.
